# Risk of osteoporosis in patients treated with direct oral anticoagulants vs. warfarin: an analysis of observational studies

**DOI:** 10.3389/fendo.2023.1212570

**Published:** 2023-09-29

**Authors:** Yumeng Liu, Xiaoping Xie, Songqi Bi, Qiong Zhang, Qingxu Song, Yang Sun, Tiecheng Yu

**Affiliations:** ^1^ Institute of Virology and AIDS Research, First Hospital of Jilin University, Jilin University, Changchun, China; ^2^ Department of Orthopedics, Orthopedics Center, First Hospital of Jilin University, Jilin University, Changchun, China

**Keywords:** direct oral anticoagulant, warfarin, osteoporosis, atrial fibrillation, meta-analysis

## Abstract

**Aims:**

Evidence on the association between the risk of new-onset osteoporosis and oral anticoagulants remains controversial. We aimed to compare the risk of osteoporosis associated with the use of direct oral anticoagulants (DOACs) with that associated with warfarin use.

**Methods:**

Studies published up to 15 March 2023 that investigated the association between the use of DOACs and warfarin and the incidence of osteoporosis were identified by online searches in PubMed, Embase, the Cochrane Library, and Web of Science conducted by two independent investigators. Random-effects or fixed-effect models were employed to synthesize hazard ratios (HRs)/relative ratios (RRs) with 95% confidence intervals (CIs) for estimating the risk of osteoporosis correlated with DOAC and warfarin prescriptions (PROSPERO No. CRD42023401199).

**Results:**

Our meta-analysis ultimately included four studies involving 74,338 patients. The results suggested that DOAC use was associated with a significantly lower incidence of new-onset osteoporosis than warfarin use (pooled HR: 0.71, 95% CI: 0.57 to 0.88, *p* < 0.001, *I* ^2^: 85.1%). Subanalyses revealed that rivaroxaban was associated with a lower risk of osteoporosis than both warfarin and dabigatran. In addition, DOACs were associated with a lower risk of developing osteoporosis than warfarin in both male and female patients, in patients with atrial fibrillation (AF), and in patients who underwent therapy for > 365 days.

**Conclusion:**

DOAC users experienced a lower incidence of osteoporosis than warfarin users. This study may give us insight into safe anticoagulation strategies for patients who are at high risk of developing osteoporosis.

**Systematic review registration:**

https://www.crd.york.ac.uk/PROSPERO, identifier CRD42023401199.

## Introduction

Warfarin, a vitamin K antagonist, is a traditional oral anticoagulant that has been used in many situations for the prevention or treatment of thromboembolic diseases or events. Warfarin can produce an anticoagulant effect by inhibiting the γ-carboxylation of coagulation factors II, VII, IX, and X (1). Studies have reported that there is a correlation between warfarin and hypocarboxylated osteocalcin, which is associated with low bone mineral density (BMD) (2, 3). Moreover, several studies indicate that long-term warfarin use is associated with reduced BMD and increased risk of developing osteoporosis and osteoporotic fractures (4–7). However, no significant association between warfarin use and osteoporosis has been identified in other studies (8, 9). Recently, direct oral anticoagulant (DOAC) therapy has been considerably expanded for clinical use, especially for the prevention of embolic phenomena in patients with atrial fibrillation (AF) (10, 11) and the treatment and prevention of venous thrombosis and pulmonary thromboembolism (12, 13). DOACs are non-vitamin K antagonists and include factor Xa inhibitors (apixaban and rivaroxaban) and thrombin inhibitors (dabigatran). The efficacy of DOACs has been described as equal or superior to that of warfarin, and patients receiving DOACs have required less anticoagulant monitoring (14–16). Moreover, many studies have demonstrated that DOAC users have a lower risk of developing osteoporosis than warfarin users (17–19). Nalevaiko et al. (20) recently indicated that patients using warfarin had a lower BMD and more degraded bone microarchitecture than patients using DOACs. In addition, it was reported that DOACs were associated with a significantly lower risk of osteoporotic fractures than warfarin use (17, 21). Our previous study suggested that the use of DOACs generated a lower risk of experiencing fractures than the use of warfarin in AF patients, particularly those with a history of osteoporosis (22). However, Lucenteforte et al. found no significant difference between the association of osteoporotic fractures with DOACs and their association with warfarin (23).

As oral anticoagulants are commonly prescribed for older patients who are vulnerable to thromboembolic events, the possible risks of osteoporosis constitute a vital clinical issue. Therefore, we conducted a meta-analysis to determine the correlation of DOACs vs. warfarin with the risk of osteoporosis by pooling the data from the included observational studies. These studies may indicate preferable anticoagulation strategies for patients at high risk of developing osteoporosis.

## Methods

The meta-analysis and systematic review were carried out in accordance with the PRISMA guidelines (24) and the MOOSE statements (**Supplementary Table 1**) (25). The meta-analysis was registered at the PROSPERO website (https://www.crd.york.ac.uk/PROSPERO/; CRD42023401199).

### Literature search strategy

Two investigators (YML and XPX) searched PubMed, Embase, Web of Science, and the Cochrane Library databases for eligible studies published before 15 March 2023. The search items included “osteoporosis”, “bone mineral density”, “direct oral anticoagulant”, “non-vitamin K antagonist”, “vitamin K antagonist”, and “warfarin”. The detailed search strategies are presented in **Supplementary Table 2**. The reference lists of all included studies and relevant reviews were also screened to identify additional studies. In addition, unpublished articles were identified from the ClinicalTrials.gov website, gray literature, and through consultation with experts in the field.

### Study selection criteria

Studies were included if they fulfilled the following inclusion criteria: (1) patients had been prescribed a DOAC or warfarin for the first time; (2) the study aimed to evaluate the association between the risk of developing osteoporosis and DOAC use compared with warfarin use; (3) the study provided sufficient data to pool the results, such as hazard ratios (HRs) or relative risks (RRs) with 95% confidence intervals (CIs); and (4) the study design was an observational study. Patients who initiated osteoporosis medication were also considered to have osteoporosis. The exclusion criteria were as follows: (1) duplicate reports; (2) case reports, editorials, reviews, and meta-analyses; and (3) animal or molecular biology studies.

Two investigators (YML and XPX) independently screened the articles by the titles and abstracts. Subsequently, the full texts were obtained to identify eligible studies. Disagreements were resolved by extensive discussion or consultation when necessary.

### Data extraction and quality assessment

The data were extracted using a standardized table. The collected data included the first author, year of publication, region, number of patients, study period, study participants, and study outcomes. For outcome data, the HRs or RRs with 95% CIs were extracted to synthesize the results. Two investigators (YML and XPX) independently conducted the extraction process. Discrepancies were resolved by discussion. Consultation with a third reviewer (TCY) was sought when necessary. Furthermore, we evaluated the quality of the included observational studies using the Newcastle–Ottawa Scale (NOS) (26). Studies with a score of eight or more were regarded as being of high quality (27).

### Data synthesis and statistical analysis

For the primary analysis, the risk of developing osteoporosis in DOAC users was compared with that in warfarin users. HRs or RRs with corresponding 95% CIs were collected to calculate pooled outcomes. Pooled HRs/RRs were presented for estimation in this meta-analysis. Heterogeneity was assessed by using the Q statistic and the *I* ^2^ statistic. Values of *p* < 0.05 and *I* ^2^ > 50% indicated significant heterogeneity across the included studies. Subsequently, random-effects models were employed to pool the results of HRs or RRs and corresponding 95% CIs for osteoporosis when heterogeneity was statistically significant. Fixed-effect models were used when heterogeneity was not statistically significant. Furthermore, we conducted subgroup meta-analyses based on individual DOACs, comparisons among each DOAC, gender, duration of therapy, and patients with AF or an unspecified condition. In addition, sensitivity analyses were performed to test whether or not the included studies had a high risk of bias and to examine the robustness of the results by removing individual studies. Publication bias was assessed using a funnel plot, Begg’s rank correlation, and Egger’s weighted regression methods. A two-tailed *p* < 0.05 was considered statistically significant. All analyses were conducted using STATA software, version 12.0 (StataCorp, TX, USA).

## Results

### Literature search

**Figure 1:**
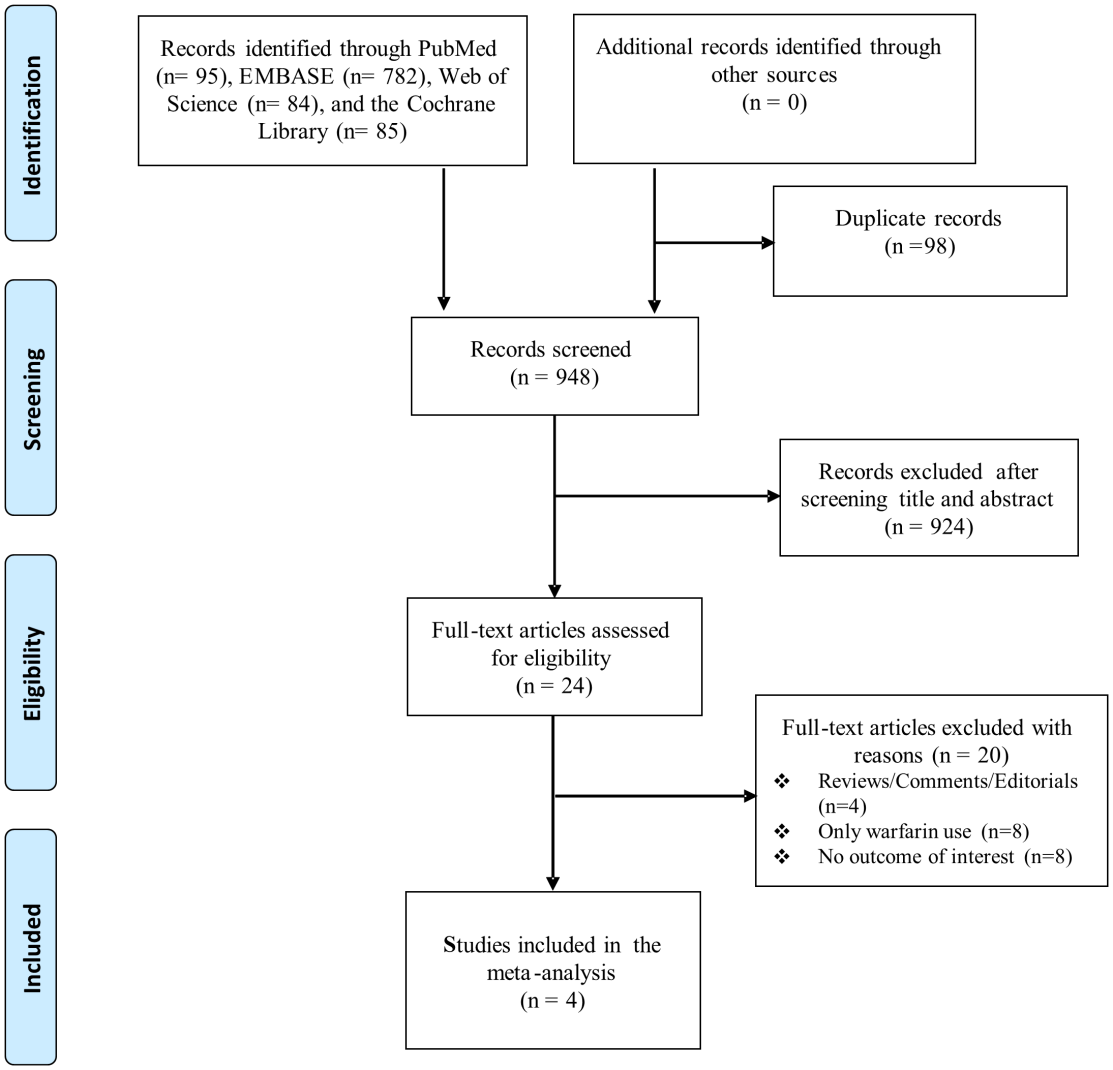
Flow diagram of study selection.

The database search identified 1,046 potentially relevant articles (**Supplementary Table 2**). A total of 98 duplicate articles were removed and 924 articles were excluded after their titles and abstracts were scanned. Subsequently, we selected the remaining potentially eligible 24 articles for full-text review, and eventually determined that four studies fulfilled the inclusion criteria; these were included in this meta-analysis (17–19, 28). The article search and selection process is summarized in the literature flow diagram (**Figure 1**).

### Study characteristics

There were four studies included in our meta-analysis (17–19, 28). Detailed study characteristics are provided in **Table 1**. These four studies were retrospective observational studies and the study duration ranged from 4 to 8 years. This meta-analysis involved 74,338 patients who had been prescribed DOACs or warfarin for the first time. Among these participants, 3,017 developed osteoporosis, of whom 1,741 were DOAC users (3.7%) and 1,276 were warfarin users (4.9%) **(** **Supplementary Table 3**). The baseline characteristics of the participants in each study are summarized in **Table 2**. The average age of these patients was over 70 years. According to the NOS guidelines, these four studies had NOS scores between eight and nine, indicating high quality (**Supplementary Table 4**).

**Table 1:** Characteristics of the four included studies.

Author (year)	Region	Sample size	Source of data	Study period	Study design (S)	Study population (P)	Exposure/comparison (I/C)	Outcome (O)	Measures
Binding et al. (2019) (17)	Denmark	37,350	The Danish National Patient Register	2013–2017	Retrospective cohort study	Patients with AF treated with OACs	DOAC vs. VKA	Initiation of osteoporosis medication	aHR
Huang et al. (2020) (18)	Taiwan	17,008 after matching*	Taiwan’s National Health Insurance Research Database	2012–2016	Retrospective cohort study	Patients with AF treated with OACs	DOAC vs. warfarin	Newly recorded osteoporosis	aHR
Patil et al. (2021) (19)	USA	1,886 after matching*	Salem Veterans Affairs Medical Center (SVAMC)	2012–2020	Retrospective single-center cohort study	Veteran patients treated with OACs	DOAC vs. warfarin	New-onset osteoporosis	aHR
Bezabhe et al. (2022) (28)	Australia	18,454 after matching	MedicineInsight program	2013–2018	Retrospective cohort study	Patients with AF treated with OACs	DOAC vs. warfarin	Newly recorded osteoporosis	aHR

AF, atrial fibrillation; DOAC, direct oral anticoagulant; OACs, oral anticoagulants; VKA, vitamin K antagonist; aHR, adjusted hazard ratio.

**Table 2:** Characteristics of participants in the included studies.

	Binding et al. (17)		Bezabhe et al. (28)				Huang et al. (18)		Patil and Hobson, (19)	
	DOAC	VKA	Dabigatran	Rivaroxaban	Apixaban	Warfarin	NOAC	Warfarin	DOAC	Warfarin
**Participants**	**25,182**	**12,168**	**1,714**	**5,871**	**5,248**	**5,621**	**8,504**	**8,504**	**943**	**943**
**Age (years), mean ± SD**	**73**	**72**	**73.1 ± 9.5**	**71.8 ± 10.0**	**73.9 ± 10.1**	**74.1 ± 10.7**	**72.0 ± 11.4**	**70.8 ± 11.9**	**70.62 ± 10.24**	**70.24 ± 10.34**
**Female subjects (%)**	**44.1**	**38.2**	**38.7**	**37.2**	**42.1**	**39.8**	**41.2**	**40.3**	**929 (1.5%)**	**921 (2.3%)**
**CHA2DS2-VASc score, mean ± SD**	**NA**	**NA**	**3.7 ± 1.7**	**3.4 ± 1.7**	**3.7 ± 1.7**	4.0 ± 1.8*	**2.4 ± 1.7**	**2.5 ± 1.7**	**3.84 ± 1.68**	3.92 ± 1.69*
Comorbidities or medical conditions, *n* (%)										
Hypertension	**NA**	**NA**	**1,258 (73.4)**	**4,122 (70.2)**	**3,780 (72.0)**	**3,990 (71.0)**	**6,465 (76.0)**	**6,579 (77.4)**	**810 (85.89)**	**817 (86.84)**
Diabetes mellitus	**3,480 (13.8)**	**1,713(14.1)**	**493 (28.8)**	**1,597 (27.2)**	**1,364 (26.0)**	**1,713 (30.5)**	**2,832 (33.3)**	**2,922 (34.4)**	**445 (47.19)**	**448 (47.51)**
Coronary artery disease	**NA**	**NA**	**421 (24.6)**	**1,550 (26.4)**	**1,507 (28.7)**	**1,794 (31.9)**	**3,769 (44.3)**	**3,851 (45.3)**	**125 (13.26)**	**137 (14.53)**
Heart failure	**436 7(17.3)**	**2,235 (18.4)**	**408 (23.8)**	**1,266 (21.6)**	**1,254 (23.9)**	**1,947 (34.6)**	**3,485 (41.0)**	**3,295 (38.8)**	**280 (29.69)**	**287 (30.43)**
Prior ischemic stroke	**4,420 (17.6)**	**1,503 (12.4)**	**344 (20.1)**	**928 (15.8)**	**928 (15.8)**	**1,257 (22.4)**	**2,662 (27.4)**	**2,509 (25.9)**	**200 (21.21)**	**215 (22.79)**
COPD	**2,468 (9.8)**	**1,114 (9.2)**	**269 (15.7)**	**883 (15.0)**	**796 (15.2)**	**1,062 (18.9)**	**1,953 (23.0)**	**2,014 (23.7)**	**495 (52.49)**	**511 (54.19)**
Medication use, *n* (%)										
Corticosteroids	**1,522 (6.0)**	**763 (6.3)**	**NA**	**NA**	**NA**	**NA**	**458 (5.4)**	**451 (5.3)**	**80 (8.48)**	**81 (8.59)**
NSAIDs	**2,365 (9.4)**	**1,947 (16.0)**	**540 (31.5)**	**2,090 (35.6)**	**1,772 (33.8)**	**1,314 (23.4)**	**2,290 (26.9)**	**2,315 (27.2)**	**510 (54.08)**	**515 (54.61)**
Statins	**10,796 (42.9)**	**5,326 (43.8)**	**973 (59.7)**	**3,355 (59.3)**	**3,073 (60.7)**	**3,186 (60.7)**	**1,699 (20.0)**	**1,865 (21.9)**	**539 (57.16)**	**531 (56.31)**
Proton-pump inhibitors	**NA**	**NA**	**925 (54.0)**	**2,925 (49.8)**	**2,735 (52.1)**	**3,018 (53.7)**	**681 (8.0)**	**671 (7.9)**	**375 (39.76)**	**375 (39.76)**

COPD, chronic obstructive pulmonary disease; DOAC, direct oral anticoagulant; NOAC, novel oral anticoagulant; NSAIDs, non-steroidal anti-inflammatory drugs; VKA, vitamin K antagonist.

CHA2DS2-VASc, congestive heart failure, hypertension, age ≥75, diabetes mellitus, prior stroke or transient ischemic attack, vascular disease, age 65–74, female.

### Meta−analysis results

**Figure 2:**
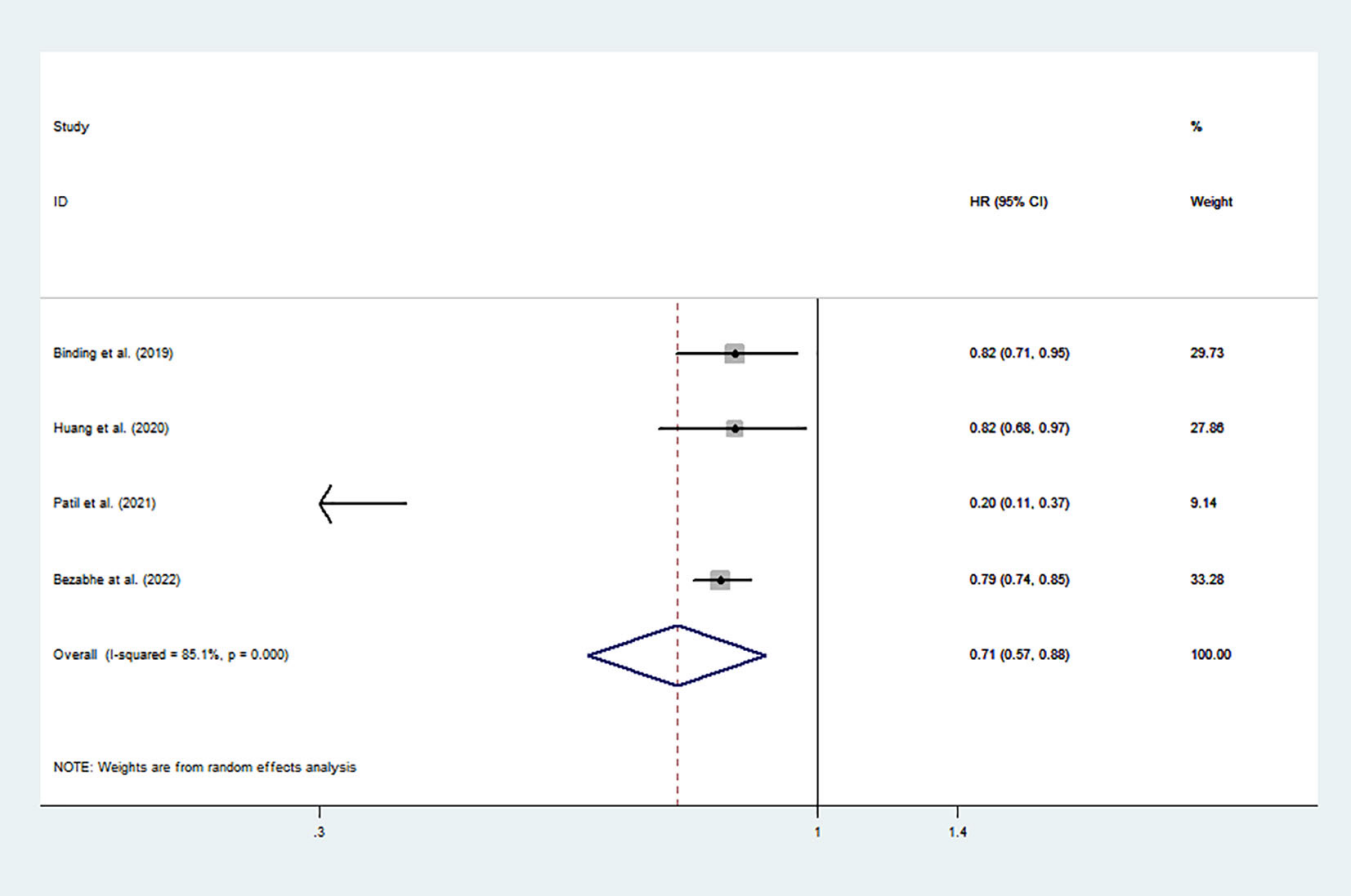
Risk of osteoporosis associated with all DOACs vs. warfarin. HR, hazard ratio; CI, confidence interval; DOACs, direct oral anticoagulants.

There were four studies involving a total of 74,338 patients included in the data analyses. The pooled results indicated that DOAC use was associated with a significantly lower risk of new-onset osteoporosis than warfarin use (pooled HR: 0.71, 95% CI: 0.57 to 0.88, *p* < 0.001, *I* ^2^: 85.1%; **Figure 2**). This result was based on a random effects model because the heterogeneity between studies was significant.

### Subgroup analyses

#### Subanalyses for individual DOACs vs. warfarin

In the subanalyses comparing each DOAC with warfarin, the results showed that rivaroxaban users tended to have a lower risk of new-onset osteoporosis than warfarin users (pooled HR: 0.74, 95% CI: 0.48 to 0.80, *p* < 0.001, *I* ^2^: 0.0%). However, there was no significant difference in the risk of osteoporosis associated with apixaban and dabigatran compared with that associated with warfarin (**Table 3**).

#### Subanalyses for comparison between individual DOAC

Two studies (18, 28) reported the risk of osteoporosis associated with each individual DOAC, and the combined results showed that in AF patients, there was a lower risk of new-onset osteoporosis associated with rivaroxaban than with dabigatran (pooled HR: 0.74, 95% CI: 0.56 to 0.96, *p* = 0.03, *I* ^2^: 74.5%). In AF patients, neither dabigatran nor rivaroxaban use was significantly associated with the risk of developing osteoporosis compared with apixaban (**Table 3**).

**Table 3:** Subgroup meta-analyses for the risk of osteoporosis in patients treated with different oral anticoagulants.

Factors for subgroup analysis	Number of studies	Pooled HR (95% CI)	*p*-value	Heterogeneity		
				χ^2^	*p*-value	*I^2^* ndex
Individual DOAC vs. warfarin						
Apixaban vs. warfarin	2 (18, 28)	0.57 (0.29 to 1.15)	0.12	6.39	0.01	84.30%
Dabigatran vs. warfarin	2 (18, 28)	0.92 (0.83 to 1.03)	0.14	1.91	0.17	47.70%
Rivaroxaban vs. warfarin	2 (18, 28)	**0.74 (0.48 to 0.80)**	<0.001	0.74	0.39	0.00%
Comparison between specific DOACs						
Apixaban vs. dabigatran	2 (18, 28)	0.61 (0.28 to 1.33)	0.21	7.36	<0.01	86.40%
Apixaban vs. rivaroxaban	2 (18, 28)	1.03 (0.91 to 1.15)	0.66	1.26	0.26	20.80%
Rivaroxaban vs. dabigatran	2 (18, 28)	**0.74 (0.56 to 0.96)**	0.03	3.92	0.05	74.50%
Gender						
Male subjects						
DOAC vs. warfarin	2 (18, 19)	**0.80 (0.72 to 0.89)**	<0.001	0.36	0.55	0.00%
Female subjects						
DOAC vs. warfarin	2 (18, 19)	**0.80 (0.74 to 0.87)**	<0.001	0.01	0.92	0.00%
Duration of therapy (days)						
>365	2 (18, 19)	**0.65 (0.50 to 0.85)**	<0.001	3.33	0.07	85.10%
<365	–	**-**	–	–	–	–
Patients included						
Atrial fibrillation	3 (17, 18, 28)	**0.80 (0.75 to 0.85)**	<0.001	0.31	0.86	0.00%
Not specified	1 (19)	0.39 (0.24 to 0.65)	<0.001	–	–	–

CI, confidence interval; DOAC, direct oral anticoagulant; HR, hazard ratio.

The meaning of the bold values can default to being a positive result. Both HR and the corresponding 95% confidence interval (95%CI) are less than 1.

#### Subanalyses for gender and the duration of therapy

Two studies (18, 19) compared the risk of osteoporosis between DOAC and warfarin in both male and female users. For the overall comparison between DOACs and warfarin, the combined results indicated that the risk of developing osteoporosis decreased in patients prescribed DOACs compared with those prescribed warfarin, regardless of gender (pooled HR: 0.80, 95% CI: 0.72 to 0.89, *p* < 0.001, *I* ^2^: 0.00% for male patients, and pooled HR: 0.80, 95% CI: 0.74 to 0.87, *p* < 0.001, *I* ^2^: 0.0% for female patients; **Table 3**). In addition, in the analyses stratified by treatment duration, DOACs were associated with a lower risk of osteoporosis than warfarin in patients who had used the medication for more than 1 year (duration of therapy > 365 days).

#### Patient subanalyses

Only patients with non-valvular AF participated in three studies (17, 18, 28), and patients’ disease statuses were not clearly stated in one study (19). The results indicated that DOACs were associated with a lower risk of new-onset osteoporosis than warfarin in patients with non-valvular AF (HR: 0.80, 95% CI: 0.75 to 0.85, *p* < 0.001, *I* ^2^: 0.0%; **Table 3**).

#### Sensitivity analysis and publication bias

After removing one study at a time in the sensitivity analyses, the risk of new-onset osteoporosis for DOAC users vs. warfarin users appeared to be stable and robust (**Supplementary Table 5**; **Supplementary Figure 1**). In this meta-analysis, there was no obvious publication bias based on the funnel plots (**Supplementary Figure 2**), Begg’s test (*p* = 0.50), or Egger’s test (*p* = 0.53).

## Discussion

This meta-analysis based on four observational studies indicated that patients prescribed DOACs were at a significantly lower risk of experiencing new-onset osteoporosis than those prescribed warfarin. This meta-analysis was based on significant heterogeneity (*I* ^2^: 85.1%). In addition, the subanalyses for each DOAC vs. warfarin revealed that rivaroxaban use was associated with a significantly lower risk of new-onset osteoporosis than warfarin and dabigatran use. In addition, DOACs tended to be associated with a lower risk of new-onset osteoporosis than warfarin in both male and female patients and in patients with non-valvular AF. Among patients treated for more than 365 days, the association between DOAC use and a lower incidence of osteoporosis appeared to be stronger than that between warfarin use and a lower incidence of osteoporosis.

Several reasons may explain why the risk of developing osteoporosis is lower in patients prescribed DOACs than in those prescribed warfarin. Three typical vitamin K-dependent proteins, namely osteocalcin, matrix Gla protein, and growth arrest-specific protein, are closely related to the maintenance of bone strength (29). Considering that vitamin K is essential for the γ-carboxylation of these proteins, warfarin could control the functions of these proteins and subsequently lead to low BMD and osteoporosis (30). Moreover, long-term warfarin exposure at clinically relevant doses was found to increase osteoclast numbers and decrease the numbers and activity levels of osteoblasts (31). In contrast, given that DOACs are oral anticoagulants based on vitamin K-independent processes, they theoretically have a lesser effect on bone health. An *in vivo* study demonstrated that patients in the dabigatran-treated group had an increased bone volume and decreased trabecular separation compared to those in the warfarin-treated group (32). In addition, an experimental study indicated that rivaroxaban treatment did not impair femur fracture healing in a rat model (33), and Gigi et al. (34) revealed that rivaroxaban inhibited osteoblast metabolism. These studies revealed that DOACs may have a protective effect on bone health. Accordingly, the risk of developing osteoporosis may decrease in DOAC users compared with warfarin users.

Recently, a cross-sectional study showed that lower BMDs and trabecular bone scores (TBSs) were seen in patients on anticoagulants than in those not on anticoagulants, which was more pronounced with warfarin use than DOACs. This conclusion was inconsistent with the findings of a study of two retrospective cohorts conducted by Bezabhe et al. (35). In addition, a study using multi-methodological data mining demonstrated an association between warfarin use (but not DOACs) and osteoporosis, regardless of gender differences (36). As we mentioned previously, long-term warfarin use may have a negative effect on BMD (4–7). Our pooled results revealed that, in patients who had received anticoagulants for more than 365 days, there was a lower incidence of osteoporosis in those who used DOACs than in those who used warfarin. Despite the patients in their study having a therapy duration of over 548 days, the conclusions of Bezabhe et al. (28) were consistent with ours. There are also many studies showing a significant association between DOAC use and a lower risk of developing fractures in AF patients than between fractures in AF patients and warfarin use. The primary outcomes included all clinical and hospitalized fractures. For instance, Lau et al. concluded that the use of dabigatran compared with warfarin was associated with a lower risk of osteoporotic fractures in patients with AF (36). Recently, a meta-analysis conducted by Huang et al. indicated that DOAC users tended to have a lower incidence of hip fractures than warfarin users (37). Our previous meta-analysis also suggested that, among AF patients, DOAC users experienced a lower risk of fracture than warfarin users, especially among those patients with a history of osteoporosis (22). We included a recent study and conducted a meta-analysis to compare the risk of new-onset osteoporosis between DOACs and warfarin for the first time. Although our results showed that the incidence of osteoporosis in DOAC users was lower than that in warfarin users, the administration of DOACs or warfarin in elderly patients should generally be based on the risk of ischemic stroke, bleeding status, and affordability rather than the risk of osteoporosis. Therefore, patients taking oral anticoagulants need physical exercise, vitamin D supplements, and calcium supplements to protect against osteoporosis and osteoporotic fractures (38).

This meta-analysis has several strengths. First, we included observational studies that involved a large number of participants and compared the risk of developing osteoporosis in patients receiving DOACs with that in those receiving warfarin based on pooled data. In addition, we analyzed the risk of osteoporosis associated with each DOAC compared with that associated with warfarin. Second, the studies included in this meta-analysis had a relatively long study duration for the emergence of osteoporosis.

This meta-analysis has several limitations. First, due to unmeasured confounders, observational studies are prone to bias. Although sophisticated analysis methods, such as propensity score matching, were employed in the included studies, unmeasured confounders remained. Second, the included observational studies were based on routinely collected electronic health records that were not designed to study osteoporosis, which may have resulted in misclassification bias in the outcomes. For example, BMD data were not available for baseline characteristics in all studies. Finally, a high degree of heterogeneity exists in this meta-analysis, which may limit the findings. Our subanalyses indicated that an individual DOAC could potentially contribute to heterogeneity. Therefore, our results cannot completely determine causality in the association between DOACs or warfarin and the risk of osteoporosis, and this conclusion should be interpreted with caution.

## Conclusion

This meta-analysis demonstrated that new DOAC users experienced a lower incidence of osteoporosis than warfarin users. Among all the oral anticoagulants, rivaroxaban was associated with a lower risk of osteoporosis than both warfarin and dabigatran. In addition, DOAC use was significantly associated with a lower risk of osteoporosis than warfarin use in both male and female patients, and in patients with non-valvular AF. Further randomized controlled trials are needed to determine the comparative risk of osteoporosis for different DOACs and warfarin to provide safe anticoagulation options for patients with risk factors for osteoporosis.

## Data availability statement

The original contributions presented in the study are included in the article/**Supplementary Material**. Further inquiries can be directed to the corresponding author.

## Author contributions

YL designed the study, conducted statistical analysis of the data, and drafted the manuscript. XX, SB, QZ, QS, and YS contributed to the writing and discussed the manuscript. TY provided critical suggestions and contributed significantly to the revision of the manuscript. All authors contributed to the article and approved the submitted version.
