# Supplementary material for: Risk of osteoporosis in patients treated with direct oral anticoagulants vs. warfarin: an analysis of observational studies
Source: Front Endocrinol (Lausanne). 2023 Sep 29;14:1212570. doi: 10.3389/fendo.2023.1212570 (PMC10570517; doi:10.3389/fendo.2023.1212570)
Supplement: Supplementary file 1 [file DataSheet_1.docx]

***Supplementary Data***

**Supplementary Table 1. The Meta-Analysis of Observational Studies in Epidemiology (MOOSE) Statement Checklist.**

**Supplementary Table 2. Summary of the search strategies.**

**Supplementary Table 3. Risk of new-onset osteoporosis with DOACs versus warfarin across four included studies.**

**Supplementary Table 4. Bias assessment of included studies using the NOS.**

**Supplementary Table 5. Sensitivity analyses: outcomes after removing individual studies.**

**Supplementary Figure 1. Sensitivity analyses for osteoporosis risks of NOACs versus warfarin through a leave-one-out approach.**

**Supplementary Figure 2. The publication bias for fracture risks of DOACs versus warfarin. Abbreviations: HR, hazard ratio.**

**Supplementary Table 2. The Meta-Analysis of Observational Studies in Epidemiology (MOOSE) Statement Checklist.**

|  | **Criteria** | **Brief description of how the criteria were handled in the meta-analysis** |
| --- | --- | --- |
|  | **Reporting of background should include** |  |
| 🗸 | Problem definition | Comparative osteoporosis risk for direct oral anticoagulants (DOACs) and warfarin remains unclear. |
| 🗸 | Hypothesis statement | The use of DOACs may be associated with a lower risk of osteoporosis compared with warfarin. |
| 🗸 | Description of the study outcomes | The primary outcome was the new-onset osteoporosis. |
| 🗸 | Types of exposure or intervention | DOACs prescription. |
| 🗸 | Type of study designs used | Observational studies were included. |
| 🗸 | Study population | Patients that firstly treated with DOAC or warfarin use. |
|  | **Reporting of search strategy should include** |  |
| 🗸 | Qualifications of searchers | The credentials of all investigators are indicated in the author list. |
| 🗸 | Search strategy, including time period included in the synthesis and keywords | Investigators conducted the literature search up to March 15, 2023. |
| 🗸 | Databases and registries searched | PubMed, Embase, the Cochrane Library, and Web of Science |
| 🗸 | Search software used, name and version, including special features | EndNote 20.0 was used to merge retrieved citations and eliminate duplications. |
| 🗸 | Use of hand searching | The reference lists of all included studies and relevant reviews were also screened to identify additional studies. Besides, unpublished articles were identified from the ClinicalTrials.gov Website, grey literature, or consult of the experts in the field. |
| 🗸 | List of citations located and those excluded, including justifications | Details of the literature review process are provided in the PRISMA flow chart. |
| 🗸 | Method of addressing articles published in languages other than English | We searched the literature without language restriction. If necessary, local scientists fluent in the original language were contacted for further information and translation. |
| 🗸 | Method of handling abstracts and unpublished studies | We included only articles published in full-text. If unpublished studies are identified, we will analyze them in a discussion section or a supplementary file further. |
| 🗸 | Description of any contact with authors | If necessary, the authors were contacted when primary outcome data was missing. If the authors did not respond, the study was excluded. |
|  | **Reporting of methods should include** |  |
| 🗸 | Description of relevance or appropriateness of studies assembled for assessing the hypothesis to be tested | Specific inclusion and exclusion criteria were described in the methods section. |
| 🗸 | Rationale for the selection and coding of data | Data extractions from each eligible study were relevant to the first author, year of publication, region, number of patients, study period, study participants, study outcomes. and possible confounding factors. |
| 🗸 | Assessment of confounding | Subgroup and sensitivity analysis were assessed to address potential confounding. |
| 🗸 | Assessment of study quality, including blinding of quality assessors; stratification or regression on possible predictors of study results | A scale explicitly addressing the quality of the study design was assessed by using the NOS. |
| 🗸 | Assessment of heterogeneity | The heterogeneity was assessed by using the Cochran *Q* test and *I^2^* for all analyses. |
| 🗸 | Description of statistical methods in sufficient detail to be replicated | All description of statistical methods, subgroup analyses, and sensitivity analyses are described in the methods section. |
| 🗸 | Provision of appropriate tables and graphics | One tables and one figures were provided primary and subgroup findings. |
|  | **Reporting of results should include** |  |
| 🗸 | Graph summarizing individual study estimates and overall estimate | Figure 2. |
| 🗸 | Table giving descriptive information for each study included | Table 1. |
| 🗸 | Results of sensitivity testing | See results section, supplementary Table 5 and supplementary Figure 1. |
| 🗸 | Indication of statistical uncertainty of findings | 95% confidence intervals and the Cochran Q test and *I^2^* were reported with all summary effect estimates. |
|  | **Reporting of discussion should include** |  |
| 🗸 | Quantitative assessment of bias | Sensitivity analyses were assessed to quantify potential biases. |
| 🗸 | Justification for exclusion | Studies that provided only abstracts were excluded. |
| 🗸 | Assessment of quality of included studies | The study quality was described in result sections. |
|  | **Reporting of conclusions should include** |  |
| 🗸 | Consideration of alternative explanations for observed results | A comprehensive list of alternative explanations was described in the discussion section. |
| 🗸 | Generalization of the conclusions | Our findings are generalizable since we included studies from different countries and outcomes reflected the real-world clinical practice. |
| 🗸 | Guidelines for future research | We make the recommendations in the discussion sections. |
| 🗸 | Disclosure of funding source | This study was funded by the National Natural Science Foundation of China (Grant number 31970090). |

**Supplementary Table 2. Summary of the search strategies**

| **Item** | **Search terms** | **Results** |
| --- | --- | --- |
| **PUBMED** | | |
| #1 | (((((((((direct oral anticoagulant[Text Word]) OR (non vitamin K antagonist oral anticoagulant[Text Word])) OR (Direct acting oral anticoagulant[Text Word])) OR (DOAC[Text Word])) OR (NOAC[Text Word])) OR (Factor Xa inhibitors[MeSH Terms])) OR (Dabigatran[MeSH Terms])) OR (Rivaroxaban[MeSH Terms])) OR (apixaban[Supplementary Concept])) OR (edoxaban[Supplementary Concept]) | 15,183 |
| #2 | (((((((((((Warfarin[MeSH Terms]) OR (Aldocumar[Text Word])) OR (Coumadin[Text Word])) OR (acenocoumarol[MeSH Terms])) OR (Nicoumalone[Text Word])) OR (sinthrome[Text Word])) OR (vitamin K antagonist[Text Word])) OR (vitamin K inhibitor[Text Word])) OR (phenprocoumon[MeSH Terms])) OR (Phenprocoumarol[Text Word])) OR (Marcoumar[Text Word]) | 26,719 |
| #3 | ((((((((Osteoporosis[MeSH Terms]) OR (Osteoporoses[Text Word])) OR (Bone loss[Text Word])) OR (Bone Density[Text Word])) OR (Bone Densities[Text Word])) OR (Bone Mineral Density[Text Word])) OR (Bone Mineral Densities[Text Word])) OR (Bone Mineral Content[Text Word])) OR (Bone Mineral Contents[Text Word]) | 151,116 |
| #4 | #1 OR #2 AND #3 | 95 |
| **EMBASE** | | |
| #1 | 'direct oral anticoagulant' OR 'non vitamin k antagonist oral anticoagulant' OR 'blood clotting factor 10a inhibitor' OR dabigatran OR rivaroxaban OR apixaban OR edoxaban | 114,137 |
| #2 | warfarin OR aldocumar OR coumadin OR 'antivitamin k' OR (vitamin AND k AND inhibitor) | 129,200 |
| #3 | osteoporosis OR osteoporoses OR (bone AND loss) OR (bone AND density) OR (bone AND mineral AND density) OR (bone AND mineral AND content) | 402,517 |
| #4 | #1 AND #2 AND #3 | 782 |
| **Web of Science** | | |
| #1 | (((((((((TS=(direct oral anticoagulant)) OR TS=(non vitamin K antagonist oral anticoagulant)) OR TS=(Direct acting oral anticoagulant)) OR TS=(DOAC)) OR TS=(NOAC)) OR TS=(Factor Xa inhibitors)) OR TS=(Dabigatran)) OR TS=(Rivaroxaban)) OR TS=(apixaban)) OR TS=(edoxaban) | 37,508 |
| #2 | ((((((((((TS=(Warfarin)) OR TS=(Aldocumar)) OR TS=(Coumadin)) OR TS=(acenocoumarol)) OR TS=(Nicoumalone)) OR TS=(sinthrome)) OR TS=(vitamin K antagonist)) OR TS=(vitamin K inhibitor)) OR TS=(phenprocoumon)) OR TS=(Phenprocoumarol)) OR TS=(Marcoumar) | 70,333 |
| #3 | (((((TS=(Osteoporosis)) OR TS=(Osteoporoses)) OR TS=(Bone loss)) OR TS=(Bone Density)) OR TS=(Bone Mineral Density)) OR TS=(Bone Mineral Content) | 433,343 |
| #4 | #1 AND #2 AND #3 | 84 |
| **The Cochrane Library** | | |
| #1 | (direct oral anticoagulant):ti,ab,kw OR (non vitamin K antagonist oral anticoagulant):ti,ab,kw OR (Factor Xa inhibitors):ti,ab,kw OR (Dabigatran):ti,ab,kw OR (Rivaroxaban):ti,ab,kw OR (apixaban):ti,ab,kw OR (edoxaban):ti,ab,kw | 4745 |
| #2 | **(Warfarin):ti,ab,kw OR (Aldocumar):ti,ab,kw OR (Coumadin):ti,ab,kw OR (acenocoumarol):ti,ab,kw OR (vitamin K antagonist):ti,ab,kw OR (vitamin K inhibitor):ti,ab,kw OR (phenprocoumon):ti,ab,kw OR (Phenprocoumarol):ti,ab,kw OR (Marcoumar):ti,ab,kw** | 6,283 |
| #3 | **(Osteoporosis):ti,ab,kw OR (Osteoporoses):ti,ab,kw OR (Bone loss):ti,ab,kw OR (Bone Density):ti,ab,kw OR (Bone Mineral Density):ti,ab,kw OR (Bone mineral content ):ti,ab,kw** | 26183 |
| #4 | #1 OR #2 | 9113 |
| #5 | #3 AND #4 | 85 |

**Supplementary Table 3. Risk of new-onset osteoporosis with DOACs versus warfarin across four included studies.**

| **Author (year)** | **Total sample size** | **DOACs** | | **Warfarin** | | **Adjusted HR (95% CI)** |
| --- | --- | --- | --- | --- | --- | --- |
|  |  | **No. of cases** | **Total No.** | **No. of cases** | **Total No.** |  |
| **Binding et al. (2019)** | **37,350** | **479** | **25,182** | **302** | **12,168** | **0.82(0.71-0.95)** |
| **Huang et al. (2020)** | **17,008** | **210** | **8504** | **328** | **8504** | **0.82(0.68-0.97)** |
| **Patil et al. (2021)** | **1,526** | **24** | **943** | **47** | **943** | **0.20(0.11-0.37)** |
| **Bezabhe at al. (2022)** | **18,454** | **1028** | **12833** | **599** | **5621** | **0.79(0.74-0.85)** |
| **Total** | **74,338** | **1,741** | **47,462** | **1,276** | **27,236** | **0.71(0.57-0.88)** |

**Abbreviations:** DOACs, direct oral anticoagulants; HR, hazard ratio; RR, relative ratio; CI, confidence interval.

**Supplementary Table 4. Bias assessment of included studies using the NOS.**

| **Author (Year)** | **Selection** | | | | **Comparability** | | **Outcome** | | | **Total NOS score** |
| --- | --- | --- | --- | --- | --- | --- | --- | --- | --- | --- |
|  | **Representativeness of the exposed cohort** | **Selection of the non exposed cohort** | **Ascertainment of exposure to implants** | **Demonstration that outcome of interest was not present at start of study** | **Study control for age, gender, and history of fracture** | **Study controls for any additional factor** | **Assessment of outcome** | **follow-up long enough for outcomes to occur** | **Adequacy of follow up of cohorts** |  |
| **Binding et al. (2019)** | **1** | **1** | **1** | **1** | **1** | **1** | **1** | **1** | **1** | **9** |
| **Huang et al. (2020)** | **1** | **1** | **1** | **1** | **1** | **1** | **1** | **1** | **1** | **9** |
| **Patil et al. (2021)** | **0** | **1** | **1** | **1** | **1** | **1** | **1** | **1** | **1** | **8** |
| **Bezabhe at al. (2022)** | **1** | **1** | **1** | **1** | **1** | **1** | **1** | **1** | **1** | **9** |

**Abbreviations:** NOS, the Newcastle-Ottawa Scale.

**Supplementary Table 5. Sensitivity analyses: outcomes after removing individual studies.**

| **Studies** | **Pooled HR (95% CI) after removing individual studies** |
| --- | --- |
| **All studies** | **0.71(0.57-0.88)** |
| **Binding et al. (2019)** | **0.62(0.43-0.88)** |
| **Huang et al. (2020)** | **0.64(0.47-0.87)** |
| **Patil et al. (2021)** | **0.80(0.75-0.85)** |
| **Bezabhe at al. (2022)** | **0.60(0.39-0.91)** |

**Abbreviations: HR, hazard ratio; CI, confidence interval.**


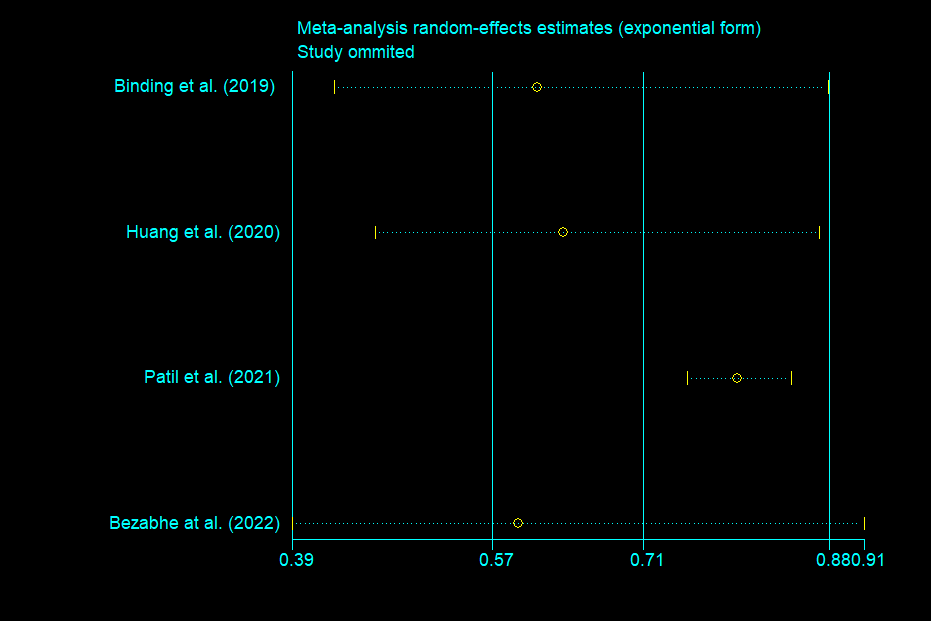


**Supplementary Figure 1. Sensitivity analyses for osteoporosis risks of NOACs versus warfarin through a leave-one-out approach. Abbreviations:** **HR, hazard ratio.**


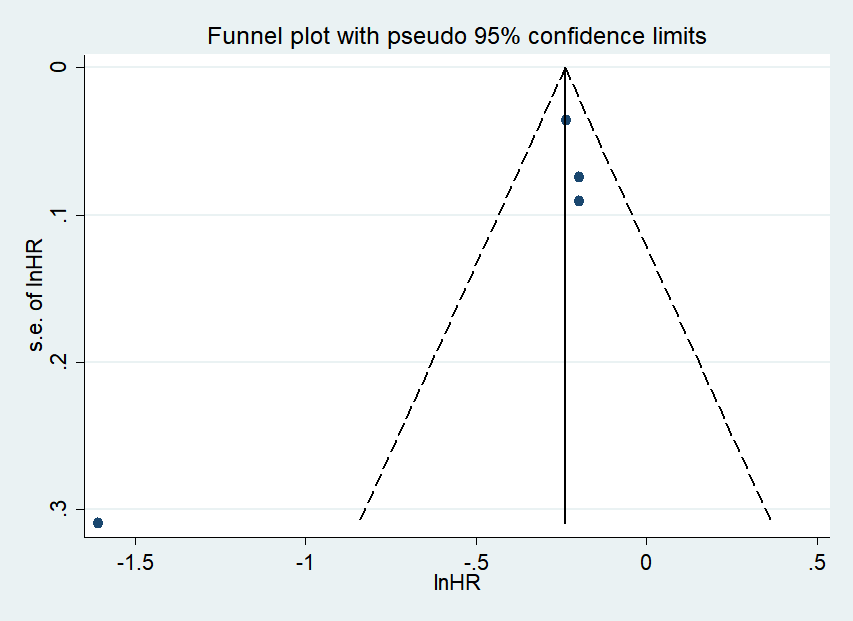


**Supplementary** Figure 2. The publication bias for fracture risks of DOACs versus warfarin. **Abbreviations:** HR, hazard ratio.
